# Comparison of the Structural Characteristics of Native Collagen Fibrils Derived from Bovine Tendons Using Two Different Methods: Modified Acid-Solubilized and Pepsin-Aided Extraction

**DOI:** 10.3390/ma13020358

**Published:** 2020-01-12

**Authors:** Haiyan Ju, Xiuying Liu, Gang Zhang, Dezheng Liu, Yongsheng Yang

**Affiliations:** 1 School of Chemistry and Chemical Engineering, Hubei Key Laboratory of Biomass Fibers and Eco-dyeing and Finishing, Wuhan Textile University, Wuhan 430073, China; juliesky2001@163.com (H.J.); liuxiuying@wtu.edu.cn (X.L.); prayerzg2019@163.com (G.Z.); 2 Hubei Key Laboratory of Power System Design and Test for Electrical Vehicle, Hubei University of Arts and Science, Xiangyang 441053, China

**Keywords:** type I collagen fibrils, acid-solubilized method, pepsin-aided extraction, aggregation structure, morphology

## Abstract

Native collagen fibrils (CF) were successfully extracted from bovine tendons using two different methods: modified acid-solubilized extraction for A-CF and pepsin-aided method for P-CF. The yields of A-CF and P-CF were up to 64.91% (±1.07% SD) and 56.78% (±1.22% SD) (dry weight basis), respectively. The analyses of both amino acid composition and sodium dodecyl sulfate-polyacrylamide gel electrophoresis (SDS-PAGE) confirmed that A-CF and P-CF were type I collagen fibrils. Both A-CF and P-CF retained the intact crystallinity and integrity of type I collagen’s natural structure by FTIR spectra, circular dichroism spectroscopy (CD) and X-ray diffraction detection. The aggregation structures of A-CF and P-CF were displayed by UV–Vis. However, A-CF showed more intact aggregation structure than P-CF. Microstructure and D-periodicities of A-CF and P-CF were observed (SEM and TEM). The diameters of A-CF and P-CF are about 386 and 282 nm, respectively. Although both A-CF and P-CF were theoretically concordant with the Schmitt hypothesis, A-CF was of evener thickness and higher integrity in terms of aggregation structure than P-CF. Modified acid-solubilized method provides a potential non-enzyme alternative to extract native collagen fibrils with uniform thickness and integral aggregation structure.

## 1. Introduction

Collagen is one of the main components of the extracellular matrix (ECM) in connective tissues. Among collagen family members, type I collagen shows the most extensive development and applications, not only because of its richness in relevant organisms, but due to having the most thorough research into its structure and properties so far [1]. It is a right-handed triple superhelical rod, which is composed of three polypeptide chains. Each of them is distinctly characterized by the repeating triplets, (Gly–X–Y)n, where X and Y are probably hydroxyproline or proline [2]. Currently, the tightest bottlenecks of natural collagen in biomedical application are lack of strength, low stability and rapid degradation. At this point, native collagen fibrils with aggregated structures have become a promising alternative, which have not only natural biological activity but also excellent physicochemical properties [3]. That is because they comprise thousands of three-stranded collagen molecules with lateral interactions, which are displaced side-by-side from one another in a quarter-staggered manner [4]. Type I collagen fibrils are of extreme importance in the regulation of cell morphology, spreading and motility, cell communication, proliferation and apoptosis [5,6,7]. In recent years, many studies have been focused on collagen fibrils reconstituted by a large number of monomeric collagen molecules in vitro. The approaches to fabricate collagen fibrils in vitro, such as self-assembly, induced-assembly and electrospinning, have also attracted widespread concerns [8,9,10]. However, due to various parameters influencing fibrillogenesis, few substantial breakthroughs of the reconstituted collagen fibrils have not been achieved in clinical applications. Besides reconstitution in vitro, the extraction of native collagen fibrils (CF) directly derived from organisms has been rarely mentioned so far [11,12].

Current studies have reported three methods of collagen extraction: neutral salt-solubilized method, pepsin-solubilized method and acid-solubilized method. The yield and physicochemical properties of collagen might change with different methods of extraction. The salt-solubilized extraction of collagen shows the lowest yield. It was reported that the yield of salt soluble collagen isolated from the marine demosponge was only 20.69 ± 1.03 mg/g (dry weight) [13]. Furthermore, it has to overcome difficulty of removing a large quantity of salts, which is used for extraction. Ample studies show that the pepsin-solubilized method has been extensively used to extract collagen with a high yield from different sources. Collagens were separately isolated from Spanish mackerel skin and Spanish mackerel bone with pepsin-solubilized extraction. The yields were 14.43 ± 0.46% and 30.01% ± 2.08% (dry weight), respectively [14]. Using the modified pepsin hydrolysis method, the yield of collagen from bester sturgeon skin was up to 63.9% ± 0.19% (dry weight) [15]. Advances have been made in terms of the structure and physicochemical properties of collagen which was exacted by conventional pepsin-aided method [16]. However, the procedures of pepsin-solubilized extraction are much more tedious and complicated, involving a lot of steps and time. Due to the enzymatic degradation of collagen, the physical and chemical properties of collagen may be decreased during the extraction [17]. To make matters worse, one might be worried about the problems of enzyme residues and inactivation, which might contaminate extracted collagen and generate low purity [18]. There are also some reports on collagen extraction from different marine animals by acid-solubilized method [19,20]. Using acid-solubilized method, collagen with different yields was extracted from the skins of marine species, with values of 4.2% for unicorn leatherjacket, 9.0% for red snapper, 25.8% for channel catfish, 41.3% for carp and 20.0% for blacktip shark, on the basis of dry weight [21]. The differences of the yields to some extent are due to the variability and living conditions of fish species.

Nevertheless, in regard to the collagen molecule, there are few reports on the methods of extracting native collagen fibrils and on comparative studies of different extraction methods. Thus, the goal of this study was to extract native collagen fibrils from bovine tendons by modified acid-solubilized extraction for A-CF and pepsin-aided method for P-CF, and then comparatively characterize A-CF and P-CF. An emphasis was placed on the aggregation structures of A-CF and P-CF utilizing a series of analytical tests. The structural comparison of A-CF and P-CF would be expected to explore a new method of extracting collagen fibrils of high purity and substantial yield without loss of their structural integrity, thereby eventually eliminating the worries about the residue and inactivation of enzyme. All the experimental results should provide reliable theoretical grounds for further applications of type I collagen fibrils.

## 2. Experimental

### 2.1. Materials

Fresh bovine tendons were bought from a local slaughterhouse in Wuhan, China. Type I collagen (COL) from bovine tendon (CAS number 9007-34-5) and pepsin from porcine stomach mucosa (3200–4500 U/mg dry matter, CAS number 9001-75-6) were supplied by Sigma Aldrich Co. (Darmstadt, Germany). Coomassie Blue R-250 and *N,N,N,N*-tetramethyl ethylene diamine (TEMED) were bought from Bio Rad Laboratories (Hercules, CA, USA). Sodium dodecyl sulphate (SDS) and the molecular weight protein markers were supplied by Fluka (Buchs, Switzerland) and New England Biolabs (Ipswich, MA, USA), respectively. The other reagents were of analytical grade.

### 2.2. Extraction of Collagen Fibrils

**Pretreatment process:** The visible muscles, fascia and fat were removed from the fresh bovine tendons. Each preprocessed tendon was cut up into 2 mm thick slices and divided into three equal parts by weight. One of the slices was freeze-dried and weighed for yield calculation (W_1_). The other two were separately used for the extractions of A-CF and P-CF. All the following extraction operations were conducted at 4 °C.

**Modified acid-solubilized extraction:** The slices were soaked into 50 mM Tris-NaCl buffer solution (100 mM NaCl, pH 7.5) for 12 h. After pouring out the buffer solution, the residue was washed by distilled water and then soaked in 0.5 M acetic acid solution in a solid/solvent proportion of 1:20 (w/v). After acidification for 72 h with stirring, the slices were centrifugally separated for 10 min at 10,000× *g*. The precipitate was crushed with a Fritsch Pulverisette mill (Idar-Oberstein, Germany). Then, the crushed residue was stirred for 48 h in 0.5 M acetic acid solution in a solid/solvent proportion of 1:20 (w/v). Then, 1.5 M ammonium sulfate was supplemented into the solution to precipitate crude collagen fibrils via salting-out, and magnetically agitated for 12 h. The resulting precipitate was collected after centrifugation (10 min, 10,000× *g*) and was re-dissolved in 0.5 M acetic acid solution under stirring. Then, the solution was dialyzed against 0.04 M NaH_2_PO_4_, 0.02 M NaH_2_PO_4_, and distilled water for 24 h, respectively. The dialysates were changed every 8 h. After dialysis, collagen fibrils (A-CF) were collected by lyophilization using a freeze dryer (Free zone 6 Liter, Kansas City, MI, USA) and weighed (W_2_) for use. 

**Pepsin-aided extraction:** The collagen fibrils (P-CF) were extracted from bovine tendons according to our previous procedures [12] with slight modifications. The first step of pretreatment was the same with A-CF. The solid/solvent ratio was 1:5 (w/v) in the step of acidification. Then pepsin was added into the solution and it was magnetically agitated for 8 h. After centrifugation (10 min, 10,000× *g*), the upper solution was collected. 1.5 M ammonium sulfate was added in the solution and magnetically stirred for 12 h. The resulting precipitate was gathered after centrifugation (10 min, 10,000× *g*) and was dissolved in 0.5 M acetic acid solution under stirring. The following dialysis and lyophilization was conducted in a similar way as A-CF. P-CF was collected and weighed (W_3_) for use. Extraction yields of collagen fibrils (dry weight) were calculated as follows:

The yield of A-CF (%) = (W_2_/W_1_) × 100%. (1)

The yield of P-CF (%) = (W_3_/W_1_) × 100%. (2)

All the samples were tested in triplicate.

### 2.3. Amino Acid Analysis

15 mg sample (A-CF, P-CF, and COL) was hydrolyzed at 110 °C in 500 µL of 6 M HCl solution for 24 h. In total, 500 µL of hydrolysate was collected and fed into Automatic Amino acid Analyzer (Hitachi 835-50, Japan) for analysis. Three samples were measured for each group, and finally, the averages were taken as the test results.

### 2.4. SDS-PAGE Measurement

According to Laemmli’s method [22], SDS-PAGE was carried out using Mini-PROTEAN Tetra Cell System (Bio-Rad Laboratories, Irvine, CA, USA). The samples (A-CF, P-CF and COL) were dissolved in 0.02 M sodium phosphate buffer (pH 7.2, SDS 1% (w/v), *β*-mercaptoethanol 5% (w/v)) solution at a final concentration of 0.03 mg/mL. Then, each was mixed with buffer solution (pH 6.8, 0.5 M Tris-HCl, glycerol 20% (v/v), SDS 4% (w/v)) at a 1:1 (v/v) proportion. The mixture was heated in boiling water for 5 min and then 8 μL was loaded into polyacrylamide gels involving 7.5% running gel and 3% stacking gel, and that underwent electrophoresis at a steady voltage of 100 V. After that, the gels were dyed with 0.05% (w/v) Coomassie Blue R-250 for 12 h. Then they were decolored by Solution I, with 10% acetic acid (v/v), 40% water (v/v) and 50% methanol (v/v) for 8 h; and Solution II, including 88% water (v/v), 5% methanol (v/v) and 7% acetic acid (v/v), for 12 h. The molecular weight markers used for electrophoretic analysis contained myosin (245 kDa), macroglobulin (180 kDa), *β*-galactosidase (135 kDa), phosphorylase B (100 kDa), bovine serum albumin (75 kDa), glutamate dehydrogenase (63 kDa), ovalbumin (48 kDa), thioredoxin reductase (35 kDa) and isomerase (25 kDa). 

### 2.5. FTIR Spectra Measurement

A 2.0 mg sample (A-CF, P-CF, and COL) was added into 80 mg KBr powder and grinded uniformly. The spectra (4000–400 cm^−1^) were gathered using FTIR (200SXV-IR, Nicolet, Thermo Electron Corporation, Waltham, MA, USA). The transmission mode was 2 cm^−1^ intervals and 30 scans each time.

### 2.6. CD Spectra Measurement

Samples (A-CF, P-CF and COL) were dissolved in 50 mM acetic acid, each at a final concentration of 0.05 mg/mL. Then, the solutions were dialyzed through 6 kDa cut-off. The dialyzed samples were re-dissolved in acetic acid solution and verified at 20 °C using the CD spectrophotometer (Model J-810CD, Jasko, Japan), and the far ultra-violet wave length was taken from 180 to 260 nm. 

### 2.7. UV–Vis Absorption Spectra Measurement

The spectra were collected in the range of 200–400 nm by the spectrophotometer (LAMBDA-25, PerkinElmer, Waltham, MA, USA). Samples were dissolved in 50 mM acetic acid at a final concentration of 1.0 mg/mL, and 1.0 mg/mL COL was the control.

### 2.8. X-Ray Diffraction Measurement

The crystallization characteristics of the samples (A-CF, P-CF and COL) were detected by XRD (XPert, Holland Philips, Amsterdam, Netherlands) with professional MDI JADE 6.0 software. The operation parameters were 40 kV and 45 mA. With Cu as the emission source, the radiation wavelength (*λ*) was 0.1541 nm. The diffraction angle was set from 5° to 50° under 2°/min scanning speed. 

### 2.9. Morphology Observations

**Scanning electron microscopy (SEM)**: Dry samples (A-CF and P-CF) of 2 mm thickness were broken by immersing in the liquid nitrogen and were sprayed t 20 kV voltage. SEM images were acquired with different magnifications by SEM (Hitachi S3000N, Tokyo, Japan).

**Transmission electron microscopy (TEM)**: 10 µL solutions (A-CF and P-CF) with the concentrations of 60 µg/mL were deposited on format carbon-coated copper grids without prior filtration. After naturally drying, the morphological structures of the samples were observed under 100 kV acceleration voltage using TEM (Tecnai G2 F20 X-TWIN, FEI Company, Hillsboro, OR, USA). 

## 3. Results and Discussion

### 3.1. Amino Acid Components

A-CF and P-CF were isolated from bovine tendons with yields of 64.91% (±1.07% SD) and 56.78% (±1.22% SD) (dry weight basis), respectively. It was mainly the pepsin digestion that cleaved the collagen fibrils to collagen molecules or smaller hydrolysates. Thus, the pepsin-aided method resulted in lower yields of type I collagen fibrils. Additionally, the yield of A-CF was higher than that of collagen by enzyme extraction reported in the literatures [15,23]. It further indicated that enzymatic hydrolysis could decrease the yield of both collagen and collagen fibrils.

The amino acid composition of type I collagen is unique, as it is rich in glycine, alanine and proline; has no cysteine; and is low in tyrosine [24]. As shown in Table 1, the amino acid compositions of A-CF, P-CF and COL are quite similar to those previously reported [25]. A-CF and P-CF contained glycine (331/1000 residues and 332/1000 residues, respectively) as the most predominant amino acid. The next were proline (135/1000 residues and 137/1000 residues, respectively) and alanine (118/1000 residues and 121/1000 residues, respectively). Hydroxyproline is the symbolic amino acid of collagen, which is not commonly present in other proteins. Moreover, its constitution may reflect the purity of a type I collagen fibril [16]. Compared with the hydroxyproline content of COL (87/1000 residues), those of A-CF and P-CF were up to 96/1000 residues and 91/1000 residues, respectively. Imino acids proline and hydroxyproline are beneficial to the triple helix stability structure of type I collagen. A-CF and P-CF had 231/1000 and 228/1000 imino/total residues respectively; those contents are higher than that of COL (223/1000 residues). The above results indicate that both A-CF and P-CF extracted from bovine tendon were type I collagen fibrils, with not only high purity but also integral triple helix structures. 

### 3.2. SDS-PAGE Pattern

**Figure 1:**
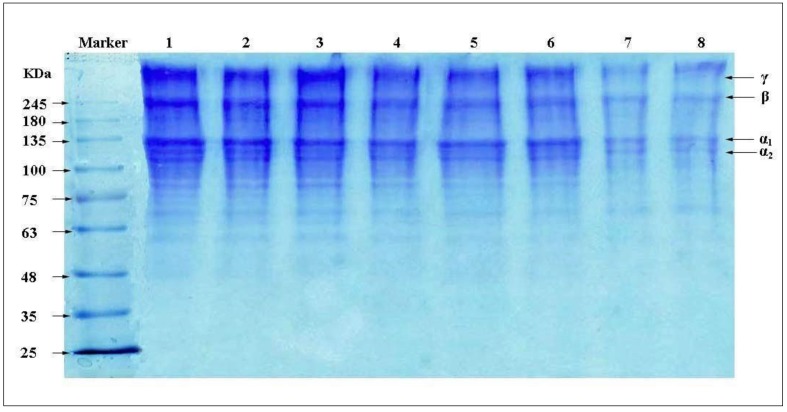
SDS-PAGE patterns of A-CF (column 1, 2, 3), P-CF (column 4, 5, 6) and COL (column 7, 8).

In the SDS-PAGE system, collagen fibrils were degraded as its intermolecular hydrogen bonds, intramolecular hydrogen bonds and covalent bonds were gradually destroyed [26]. The subunit compositions of A-CF and P-CF from bovine tendon are visualized in the electrophoretogram (Figure 1). Both A-CF and P-CF showed two different α-chains (α1 and α2) with the molecular weights (Mw) 100 and 135 kDa; *β*-chains (dimmers of the α-chains) with the Mw 245 kDa; and *γ*-chains (trimers of the α-chains). Subunit fragments with Mw lower than α-chains are hardly observable in the electrophoretogram of A-CF and P-CF. The above results are in accordance with both COL (Figure 1, column 7, 8) and type I collagen reported by Muyonga [27], which implies that the main component of both A-CF and P-CF is type I collagen, coinciding with the results mentioned in amino acid component. Additionally, the colors of β and γ stripes of A-CF are distinctly deeper than those of P-CF in the electrophoretogram. That is because of the higher Mw and more intact aggregation structure of A-CF, which leads to more subunit fragments reduced by SDS-PAGE system.

### 3.3. FTIR Spectra

**Figure 2:**
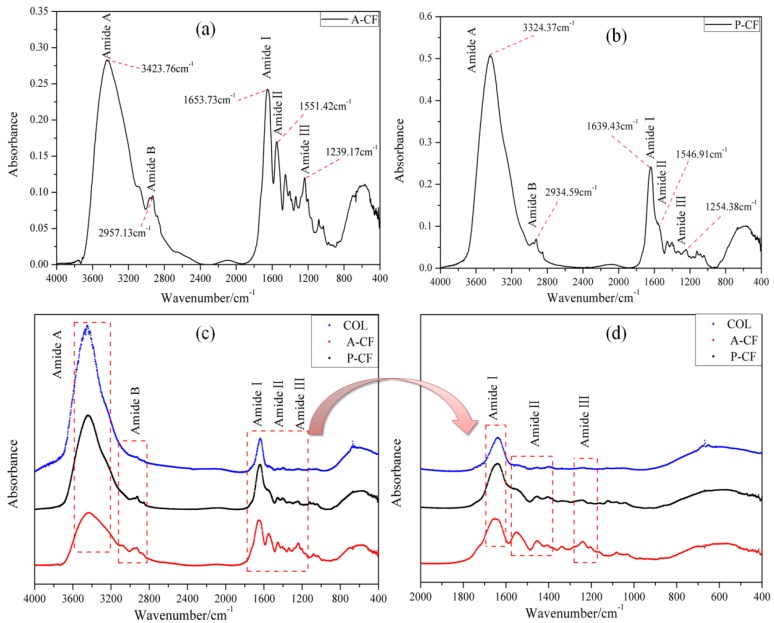
FT-IR spectra of A-CF, P-CF and COL. (**a**) A-CF; (**b**) P-CF; (**c**) COL, A-CF and P-CF (4000–400 cm^−1^); and (**d**) COL, A-CF and P-CF (2000–400 cm^−1^).

FTIR spectra are widely used to investigate the secondary structures, including the functional groups and chemical bonds, of collagen [28]. FTIR spectra of A-CF and P-CF from bovine tendons with COL as the control are shown in Figure 2, and the spectral peak identifications [29] are interpreted in Table 2. The major peaks, such as the Amide I, Amide II and Amide III in the spectra of A-CF and P-CF from bovine tendon conformed to the characteristics of type I collagen [30]. According to the description of Andrews [31], a free N–H stretching vibration of the polypeptide chains usually exists in the range of 3440–3400 cm^−1^, and when it is associated with hydrogen bond, its position would be shifted to lower frequencies. The N–H stretching vibrations of A-CF and P-CF separately appeared at 3423.76 cm^−1^ and 3324.57 cm^−1^, both cases with high peak intensity. There was a relatively weak absorption peak in both A-CF and P-CF at 2957.13 cm^−1^ and 2934.59 cm^−1^, respectively, because of the C–N stretching vibration named Amide B [32]; that is consistent with that recorded by Kunii, et al. [33]. Amide I and Amide II bands of collagen were associated with α-helix, *β*-folding, random crimp coil and superposition. The Amide I bands of A-CF and P-CF were detected at 1653.73 cm^−1^ and 1639.43 cm^−1^, respectively, which was due to the stretching vibration of carboxide groups or hydrogen bond coupled with carboxyl. Furthermore, it was found that due to N–H and –CH_2_ bending vibration as the typical absorption of type I collagen, the Amide II bands of A-CF and P-CF were separately located at 1551.42 cm^−1^, 1546.91 cm^−1^, 1254.38 cm^−1^ and 1239.17 cm^−1^.

Although the positions of Amide II band and Amide III were similar to each other, they were really critically distinguishing from the intensity of the absorption peaks. The A-CF showed that the secondary structure of A-CF extracted with acid-solubilized method is more prominent and complete than that of P-CF. The ratio of infrared absorptance (R = A_1239_/A_1453_) could indicate whether collagen’s biological activity exists or not. When the R value is close to 1, collagen could be considered to have intact triple helix structure, which is a symbol of its natural biological activity [34]. According to the data in Figure 2, it was calculated that the R values of both A-CF and P-CF were 0.99, which indicated that A-CF and P-CF maintained the integrity of type I collagen natural structure. 

### 3.4. CD Spectroscopy

**Figure 3:**
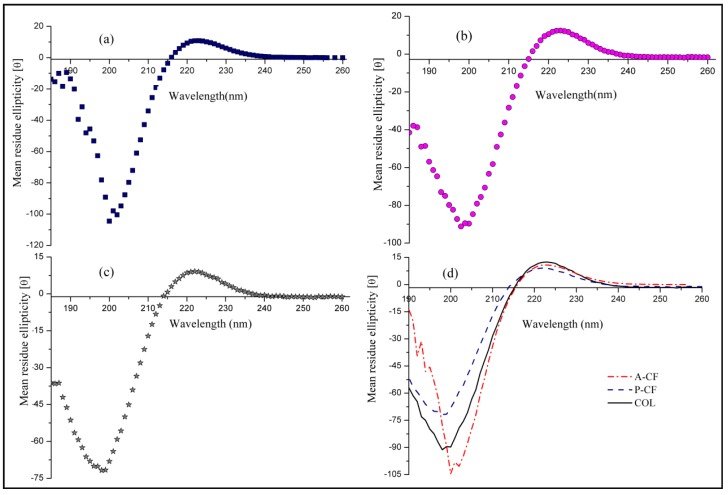
CD spectra of A-CF (**a**), P-CF (**b**) and COL (**c**); and A-CF, P-CF and COL (**d**) in the wavelength range of 185–260 nm at 20 °C.

CD spectrum was applied to further examine the triple helix structure of A-CF and P-CF. Collagen usually displays a negative minimum peak at 195–198 nm on account of the magnetic π–π* transition and a positive peak around 220 nm contributed by the electric n–π* transition [35]. When the negative peak moves to low wave or the positive peak collapses, it means that the native triple helix structure of collagen may be destroyed. As a result, the native biological activity of collagen is undermined to certain extent, which leads to a severely limit on the applications of collagen products [36]. Figure 3 shows CD spectra of A-CF, P-CF and Sigma I collagen in the range of 190–240 nm at 20 °C. Both of A-CF and P-CF exhibited specific positive and negative absorption peaks that were highly similar to Sigma I collagen. The positive and negative peaks of A-CF were at 223 and 198 nm respectively, both of which were attributed to the unique triple helices of type I collagen. Calculated by analysis software built in CD spectrophotometer, the alpha spiral degree (*f_α_*) values of A-CF, P-CF and Sigma I collagen at 208 nm were 30.14%, 28.69% and 30.13% respectively. Apparently, the *f_α_* value of A-CF is closer to that of Sigma I collagen than P-CF, which suggested that A-CF retained more intact triple helix structure than P-CF, and it may actually be more beneficial for biomedical applications. 

### 3.5. UV–Vis Absorption Spectra

**Figure 4:**
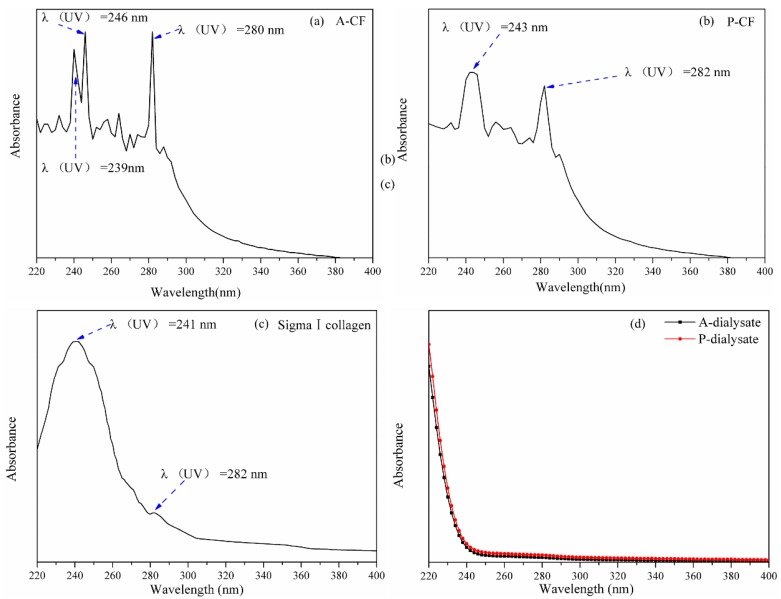
The UV–Vis spectra of A-CF (**a**), P-CF (**b**) and Sigma I collagen (**c**), and the dialysates (**d**) of A-CF and P-CF.

Collagen fibrils comprise thousands of collagen molecules through covalent cross linkages and lateral aggregation, while collagen possesses abundant chromophores, such as –COOH, >C=O and –CONH–, and guanidyl groups on the side chains to produce absorption spectra in the UV–Vis range (Figure 4). Type I collagen generally shows one absorption peak at 230 nm mainly contributed by the n–π* transition of carbonyl groups from peptide bonds, and another unique absorption peak nearby 280 nm due to the conjugated double bond from its side chains involving aromatic amino acids such as tryptophan, tyrosine and phenylalanine. As shown in the Figure 4c, Sigma I collagen molecules showed two main absorption peaks, respectively, at 241 and 282 nm, which is similar to what is reported in the literature [37]. However, A-CF showed several serrated absorption peaks, such as bimodal distribution at 239 and 246 nm, and a very sharp peak at 280 nm (Figure 4a). Similarly, P-CF had the serrated absorption peaks, especially located at 243 and 282 nm (Figure 4b). This could be due to specific difference between the aggregation structure of collagen fibrils and the molecular structure of collagen. A-CF and P-CF with aggregation structure comprised ample collagen molecules stabilized by intermolecular amide bonds, salt chemical bonds and hydrogen bonds. Thus, compared with a single collagen molecule, both A-CF and P-CF owned far higher chromophore density which contributed to serrated absorption peaks. On the other hand, the overall absorption intensity of P-CF is obviously lower than that of A-CF. This illustrates that there could be more intact aggregation structure of A-CF. In addition, it was seen that there was no characteristic peak in the dialysates of both A-CF and P-CF (Figure 4d), which indicated that collagen fibrils, either extracted by acid-solubilized method or pepsin-aided method, maintained intact aggregation structure and high purity. The above results not only reveal specific aggregation structure of A-CF and P-CF, but also verify that A-CF by acid-solubilized extraction remained more intact aggregation structure than P-CF.

### 3.6. X-Ray Diffraction

**Figure 5:**
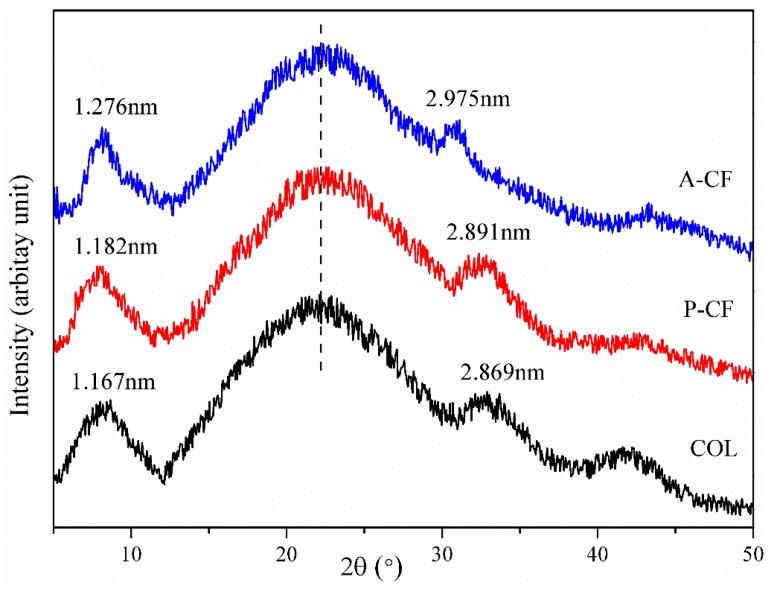
X-ray diffraction patterns of A-CF, P-CF and COL.

The spatial structure of the triple helices and collagen crystallinity could be represented by the XRD diffraction pattern [38]. In Figure 5, A-CF and P-CF show similar peak shapes with COL. Both of them exhibit a dominant sharp peak at about 9° (diffraction angle, 2θ), which is the representative feature of collagen. This peak corresponds to the diameter of the triple helix structure and represents a repeat distance (d value), which was calculated according to the Bragg equation d(Å) = *λ*/2sin*θ* (where *λ* is the X-ray wavelength (1.54 Å) and *θ* is the Bragg diffraction angle). The d values of A-CF, P-CF and COL were 1.276, 1.182 and 1.167 nm, respectively. The second peak at about 22.5° was broad and corelative to the distance between amino acid residues along the helix of collagen. Likewise, a weaker peak was observed at about 32° in the X-ray pattern of A-CF and P-CF. The d values of A-CF and P-CF were 2.975 and 2.891 nm, respectively. This may imply that the helical structures of A-CF and P-CF have almost no significant differences in the repeat separation between amino acid residues. Additionally, there was a smaller peak at about 42°. It could be formed by lyophilization during the sample preparing process. By contrast, the plots reveal that the triple helices and crystallinity of collagen fibrils were not impaired by using either acid-solubilized method or pepsin-aided extraction.

### 3.7. Morphology

**Figure 6:**
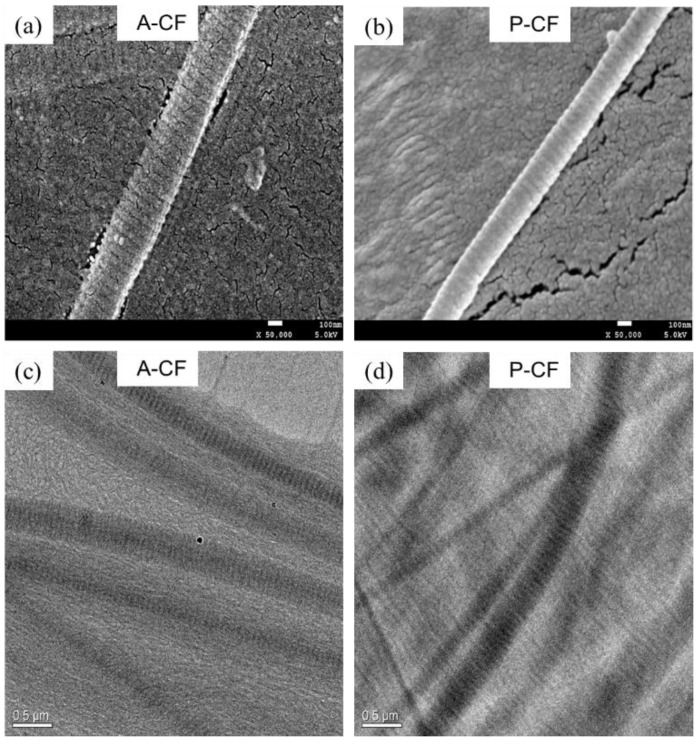
Microstructures of A-CF and P-CF. (**a**,**b**) SEM images of individual collagen fibrils. (**c**,**d**) TEM images of collagen fibrils dispersed in solution at the concentration of 60 µg/mL.

Figure 6 shows the microstructures of A-CF and P-CF observed by SEM and TEM. The D-periodicities of both A-CF and P-CF are clearly shown in Figure 6a,b, and the diameters of A-CF and P-CF are approximately 386 and 282 nm, respectively. Meanwhile, D-periodicity of collagen fibrils plays an important role in the mechanical and biological functions of collagenous matrix [39]. In the light of the 1/4 staggered model of Schmitt hypothesis [40], collagen fibrils are assembled with a great mass of tropocollagen molecules, which are in a manner of parallel staggered areas with less than full protein coverage and overlapping areas with full protein coverage. The D-periodicity of type I collagen reported is about 67 nm for wet tissues and 64 nm for air-dried samples [41]. Although A-CF is longer in diameter than P-CF, the average D-periodicity value of A-CF (66.9 nm) was similar to that of A-CF (67.3 nm). This indicates that extraction methods may influence the diameter of collagen fibrils to a certain degree, but mildly effect the D-periodicity of collagen fibrils. In Figure 6c,d, collagen fibrils were regularly dispersed in solution, but A-CF was more uniform in thickness than P-CF. This could result from the degradation of collagen fibrils by enzymolysis during the pepsin-aided extraction process. This further suggests that acid-solubilized method could be an alternative for extracting collagen fibrils with uniform thickness.

## 4. Conclusions

As a crucial natural polymer, collagen is increasingly significant in the field of biomedical materials. A-CF and P-CF were successfully extracted from bovine tendons using the acid-solubilized extraction and conventional pepsin-aided methods, respectively. The yields were 64.91% (±1.07% SD) and 56.78% (±1.22% SD) (dry weight basis) respectively. The results further verified that enzymatic hydrolysis could decrease the yields of both collagen and collagen fibrils. A-CF and P-CF were type I collagen fibrils verified by SDS-PAGE and amino acid composition analysis. Both of them retained intact crystallinity and integrity of type I collagen’s natural structure. However, A-CF showed more intact aggregation structure than P-CF by UV–Vis observation. The diameters of A-CF and P-CF were about 386 and 282 nm, respectively. Although both A-CF and P-CF were theoretically concordant with the Schmitt hypothesis, A-CF exhibited evener thickness and higher integrity in terms of aggregation structure than P-CF. The results above suggest that extraction methods have a great influence on the yield, diameter, thickness and aggregation structure of collagen fibrils. As a result of the intense degradation of collagen fibrils by pepsin, the pepsin-aided method led to unevenness and a lower yield of type I collagen fibrils. In contrast, the modified acid-solubilized method could be a potential non-enzyme alternative to extract native collagen fibrils with uniform thickness and integral aggregation structure.

## Figures and Tables

**Table 1:** Amino acid compositions of A-CF, P-CF and COL (residues/1000 amino acid residues).

Amino Acid	A-CF	P-CF	COL
Threonine	16	17	16
Serine	29	27	30
Glutamic acid	69	69	65
Glycine	331	332	330
Alanine	118	121	119
Valine	18	20	21
Methionine	8	6	6
Proline	135	137	136
Leucine	24	22	25
Tyrosine	6	7	4
Lysine	23	24	28
Histidine	5	6	5
Arginine	51	50	52
Hydroxyproline	96	91	87
Phenylalanine	11	10	11
Hydroxylysine	7	9	7
Isoleucine	10	11	12
Aspartic acid	43	41	46
Imino acids *	231	228	223

* Imino acids include Proline and Hydrolxyproline.

**Table 2:** FTIR band assignments of the A-CF and P-CF.

Region	Wavenumber (cm^−1^)		Characteristic
	A-CF	P-CF	
Amide A	3323.76	3324.57	N–H stretch, coupled with hydrogen bonding
Amide B	2957.13	2934.59	C–N stretch
	2878.45	2893.11	CH_2_ symmetric and asymmetric stretch
Amide I	1653.73	1639.43	C=O stretch, coupled with hydrogen bonding
Amide II	1551.42	1546.91	N-H bend coupled with C-N stretch
	1453.18	1447.80	CH_2_ bend
	1402.76	1398.45	COO^−^ symmetric stretch
Amide III	1239.17	1254.38	CH_2_ wagging of proline
	1082.52	1036.77	C–O stretch/C–N–C stretch

## References

[1] Stamov D.R., Nguyen T.A.K., Evans H.M., Pfohl T., Werner C., Pompe T. (2011). The impact of heparin intercalation at specific binding sites in telopeptide-free collagen type I fibrils. Biomaterials.

[2] Sun L.L., Hou H., Li B.F., Zhang Y. (2017). Characterization of acid- and pepsin-soluble collagen extracted from the skin of Nile tilapia. Int. J. Biol. Macromol..

[3] Baniasadi M., Minary-Jolandan M. (2015). Alginate-collagen fibril composite hydrogel. Materials.

[4] Kim T., Sridharan I., Ma Y., Zhu B., Chi N., Kobak W., Rotmensch J., Schieber J.D., Wang R. (2016). Identifying distinct nanoscopic features of native collagen fibrils towards early diagnosis of pelvic organ prolapse. Nanomed. Nanotechnol..

[5] Liu X.H., Dan W.H., Ju H.Y., Dan N.H., Gong J.X. (2015). Preparation and evaluation of a novel pADM-derived micro- and nano-electrospun collagen membrane. RSC Adv..

[6] Pezzoli D., di Paolo J.P., Kumra H., Fois G., Candiani G., Reinhardt D.P., Mantovani D. (2018). Fibronectin promotes elastin deposition, elasticity and mechanical strength in cellularised collagen-based scaffolds. Biomaterials.

[7] Böhm S., Strauß C., Stoiber S., Kasper C., Charwat V. (2017). Impact of source and manufacturing of collagen matrices on fibroblast cell growth and platelet aggregation. Materials.

[8] Shen L.R., Bu H.H., Yang H., Liu W.T., Li G.Y. (2018). Investigation on the behavior of collagen self-assembly in vitro via adding sodium silicate. Int. J. Biol. Macromol..

[9] Kim J.Y., Kim J.I., Park C.H., Kim C.S. (2019). Design of a modified electrospinning for the in-situ fabrication of 3D cotton-like collagen fiber bundle mimetic scaffold. Mater. Lett..

[10] Sizeland K.H., Hofman K.A., Hallett I.C., Martin D.E., Potgieter J., Kirby N.M., Hawley A., Mudie S.T., Ryan T.M., Haverkamp R.G. (2018). Nanostructure of electrospun collagen: Do electrospun collagen fibers form native structures?. Materialia.

[11] Aladin D.M.K., Cheung K.M.C., Ngan A.H.W., Chan D., Leung V.Y.L., Lim C.T. (2010). Nanostructure of collagen fibrils in human nucleus pulposus and its correlation with macroscale tissue mechanics. J. Orthop. Res..

[12] Ju H.Y., Wu N., Dan W.H., Dan N.H. (2012). Network structure and thermotropic property of type I collagen fibrils. J. Soc. Leath. Tech. Chem..

[13] Pallela R., Bojja S., Janapala V.R. (2011). Biochemical and biophysical characterization of collagens of marine sponge, Ircinia fusca (Porifera: Demospongiae: Irciniidae). Int. J. Biol. Macromol..

[14] Li Z.R., Wang B., Chi C.F., Zhang Q.H., Gong Y.D., Tang J.J., Luo H.Y., Ding G.F. (2013). Isolation and characterization of acid soluble collagens and pepsin soluble collagens from the skin and bone of Spanish mackerel (Scomberomorous niphonius). Food Hydrocoll..

[15] Meng D., Tanaka H., Kobayashi T., Hatayama H., Zhang X., Ura K., Yunoki S., Takagi Y. (2019). The effect of alkaline pretreatment on the biochemical characteristics and fibril-forming abilities of types I and II collagen extracted from bester sturgeon by-products. Int. J. Biol. Macromol..

[16] Huang Y., Huang B., Shiau C., Chen H. (2011). Isolation and characterization of acid and pepsin-solubilized collagens from the skin of balloon fish. Food Hydrocoll..

[17] Malaspina D.C., Szleifer I., Dhaher Y. (2017). Mechanical properties of a collagen fibril under simulated degradation. J. Mech. Behav. Biomed..

[18] Chuaychan S., Benjakul S., Kishimura H. (2015). Characteristics of acid- and pepsin-soluble collagens from scale of seabass (Lates calcarifer). Food Sci. Technol..

[19] Skierka E., Sadowska M. (2007). The influence of different acids and pepsin on the extractability of collagen from the skin of Baltic cod (Gadus morhua). Food Chem..

[20] Sinthusamrana S., Benjakul S., Kishimura H. (2013). Comparative study on molecular characteristics of acid soluble collagens from skin and swim bladder of seabass (Lates calcarifer). Food Chem..

[21] Vallejos N., González G., Troncoso E., Zúñiga R.N. (2014). Acid and Enzyme-Aided Collagen Extraction from the Byssus of Chilean Mussels (Mytilus Chilensis): Effect of Process Parameters on Extraction Performance. Food Biophys..

[22] Laemmli U.K. (1970). Cleavage of structural proteins during assembly of head of bacteriophage T4. Nature.

[23] Vidal A.R., Duarte L.P., Schmidt M.M., Cansian R.L., Fernandes I.A., Mello R.O., Demiate I., Dornelles R.C.P. (2020). Extraction and characterization of collagen from sheep slaughter by-products. Waste Manage.

[24] Li J., Wang M.C., Qiao Y.Y., Tian Y.Y., Liu J.H., Qin S., Wu W.H. (2018). Extraction and characterization of type I collagen from skin of tilapia (Oreochromis niloticus) and its potential application in biomedical scaffold material for tissue engineering. Process Biochem..

[25] Jongjareonrak A., Benjakul S., Visessanguan W., Nagai T., Tanaka M. (2005). Isolation and characterisation of acid and pepsin-solubilised collagens from the skin of Brownstripe red snapper (Lutjanus vitta). Food Chem..

[26] Abdollahi M., Rezaei M., Jafarpour A., Undeland I. (2018). Sequential extraction of gel-forming proteins, collagen and collagen hydrolysate from gutted silver carp (Hypophthalmichthys molitrix), a biorefinery approach. Food Chem..

[27] Muyonga J.H., Cole C.G.B., Duodu K.G. (2004). Fourier transform infrared (FTIR) spectroscopic study of acid soluble collagen and gelatin from skins and bones of young and adult Nile perch (Lates niloticus). Food Chem..

[28] Song W., Markel D.C., Wang S., Shi T., Mao G., Ren W. (2012). Electrospun polyvinyl alcohol-collagen-hydroxyapatite nanofibers: A biomimetic extracellular matrix for osteoblastic cells. Nanotechnology.

[29] Ding C., Zhang M., Li G. (2015). Preparation and characterization of collagen/hydroxypropyl methylcellulose (HPMC) blend film. Carbohyd. Polym..

[30] Kandamchira A., Selvam S., Marimuthu N., Kalarical J.S., Fathima N.N. (2013). Influence of functionalized nanoparticles on conformational stability of type I collagen for possible biomedical applications. Mater. Sci. Eng. C.

[31] Andrews M.E., Murali J., Muralidharan C., Madhulata W., Jayakumar R. (2003). Interaction of collagen with corilagin. Colloid Polym. Sci..

[32] He L., Mu C., Shi J., Shi B., Zhang Q., Lin W. (2011). Modification of collagen with a natural cross-linker, procyanidin. Int. J. Biol. Macromol..

[33] Kunii S., Morimoto K., Nagai K., Saito T., Sato K., Tonomura B. (2011). Actinidain-hydrolyzed type I collagen reveals a crucial amino acid sequence in fibril formation. J. Biol. Chem..

[34] Plepis A.M.G., Goissis G., Das-Gupta D.K. (1996). Dielectric and pyroelectric characterization of anionic and native collagen. Polym. Eng. Sci..

[35] Yang L., Fitié C.F.C., Werf K.O., Bennink M.L., Dijkstra P.J., Feijen J. (2008). Mechanical properties of single electrospun collagen type I fibers. Biomaterials.

[36] Zeugolis D.I., Khew S.T., Yew E.S.Y., Ekaputra A.K., Tong Y.W., Yung L.L. (2008). Electro-spinning of pure collagen nano-fibres-Just an expensive way to make gelatin?. Biomaterials.

[37] Bharathy H., Fathima N.N. (2017). Exploiting oleuropein for inhibiting collagen fibril formation. Int. J. Biol. Macromol..

[38] Kolanthai E., Sindu P.A., Khajuria D.K., Veerla S.C., Kuppuswamy D., Catalani L.H. (2018). Graphene oxide—A tool for the preparation of chemically crosslinking free alginate-chitosan-collagen scaffolds for bone tissue engineering. ACS Appl. Mater. Interfaces.

[39] Yousefi M., Ariffin F., Huda N. (2017). An alternative source of type I collagen based on by-product with higher thermal stability. Food Hydrocoll..

[40] Schmitt F.O., Gross J., Highberger J.H. (1955). Tropocollagen and the properties of fibrous collagen. Exp. Cell. Res..

[41] Stinson R.H., Sweeny P.R. (1980). Skin collagen has an unusual d-spacing. Biochim. Biophys. Acta.

